# Regimen-Specific Population Pharmacokinetics of Isoniazid with and Without Rifamycin: A Bayesian Modeling Analysis of 6H, 3HR, and 3H2P2 Regimens

**DOI:** 10.3390/pharmaceutics18080937

**Published:** 2026-07-30

**Authors:** Zhipeng Li, Xiao Xiao, Chunhua Xu, Yiyun Liu, Lexian Gu, Xuliang Li, Xin Shen, Yi Hu

**Affiliations:** 1 Shanghai Municipal Center for Disease Control and Prevention, Shanghai 200336, China; lizhipeng@scdc.sh.cn (Z.L.); xiaoxiao@scdc.sh.cn (X.X.); liuyiyun@scdc.sh.cn (Y.L.); 2 Fengxian District Center for Disease Control and Prevention, Fengxian District, Shanghai 201499, China; jf57193451@126.com; 3 Department of Epidemiology, School of Public Health and Key Laboratory of Public Health Safety, Fudan University, Shanghai 201203, China; 25211020009@m.fudan.edu.cn (L.G.);

**Keywords:** isoniazid, rifamycin, pharmacokinetics, Bayesian modeling

## Abstract

**Background:** Isoniazid (INH) remains a cornerstone of tuberculosis (TB) prevention and treatment, administered either as monotherapy or in combination with rifamycin-containing regimens. In this study, the preventive regimens analyzed included 6 months of daily INH monotherapy (6H), 3 months of daily INH plus rifampicin (3HR), and 3 months of twice-weekly INH plus rifapentine (3H2P2). Despite the adoption of shorter-course regimens, substantial inter-individual variability (IIV) in INH exposure persists, potentially impacting both therapeutic efficacy and toxicity. A quantitative, regimen-specific characterization of INH pharmacokinetics is therefore critical to support model-informed dosing strategies. **Methods:** Population pharmacokinetic models were developed separately for INH administered as 6H, 3HR, and 3H2P2. The models characterized absorption, clearance, and IIV of INH, while accounting for co-administered rifamycin. The effects of N-acetyltransferase 2 (NAT2) acetylator phenotype and relevant clinical covariates were systematically evaluated. Model performance was assessed using goodness-of-fit diagnostics, posterior predictive checks, and visual predictive checks. Population pharmacokinetic models were developed using a Bayesian nonlinear mixed-effects framework implemented in Stan through CmdStanR version 0.9.0, with additional data processing, statistical summaries, and visualization performed using R version 4.2.3. **Results:** INH pharmacokinetics were adequately described by regimen-specific models, revealing distinct differences in absorption and variability across regimens. Typical INH apparent oral clearance (CL/F) estimates were 21.83 L/h for 6H, 26.32 L/h for 3HR, and 25.98 L/h for 3H2P2. NAT2 phenotype was a major determinant of INH clearance across regimens: compared with intermediate acetylators, slow acetylators showed 35.4% lower CL/F, whereas fast acetylators showed 48.4% higher CL/F, indicating higher INH exposure in slow acetylators and lower exposure in fast acetylators. Body weight also influenced INH pharmacokinetics. Co-administration with rifampicin or rifapentine influenced INH pharmacokinetics in a manner consistent with reduced exposure in rifamycin-containing regimens, particularly among fast acetylators. **Conclusions:** Regimen-specific population pharmacokinetic modeling elucidated clinically relevant differences in INH exposure across commonly used preventive and treatment regimens. These findings highlight the importance of accounting for regimen- and genotype-specific effects when optimizing INH dosing and provide a quantitative framework for future model-informed precision dosing approaches in TB care.

## 1. Introduction

Isoniazid (INH) is a key component of tuberculosis (TB) prevention and treatment, administered either as monotherapy or in combination with rifamycin-containing regimens such as rifampicin (3HR) and rifapentine (3H2P2) [1,2]. Although shorter-course combination regimens have improved adherence, substantial inter-individual variability (IIV) in INH exposure persists and may affect both efficacy and toxicity [3,4]. Quantitative characterization of INH pharmacokinetics across commonly used regimens is therefore essential to support model-informed dosing strategies.

INH pharmacokinetics are characterized by rapid absorption and hepatic metabolism, with clearance strongly influenced by N-acetyltransferase 2 (NAT2) genetic polymorphisms [5,6]. Slow acetylators typically exhibit higher systemic exposure, whereas fast acetylators show lower exposure. While the influence of NAT2 on INH disposition is well established, the consistency and magnitude of this effect across different dosing regimens and in the presence of rifamycin remain incompletely defined. Rifampicin and rifapentine differ in dosing frequency, enzyme induction potential, and food-related bioavailability, which may contribute to regimen-specific differences in INH exposure and variability [7,8].

To date, few studies have systematically examined regimen-specific differences in isoniazid (INH) pharmacokinetics across 6H, 3HR, and 3H2P2 regimens while considering NAT2 acetylator phenotype and the effects of co-administered rifamycins [9,10,11]. Regimen-specific modeling is important because variations in dosing frequency, co-administered rifamycins, and sampling schedules may influence INH absorption, clearance, and variability. Such differences may be masked if data are pooled across regimens without accounting for regimen-specific structural or covariate effects. The study evaluated three tuberculosis treatment regimens: 6 months of daily isoniazid monotherapy (6H), 3 months of daily isoniazid plus rifampicin (3HR), and 3 months of twice-weekly isoniazid plus rifapentine (3H2P2). Given differences in dosing frequency, co-administered rifamycins, and potential regimen-specific pharmacokinetic variability, we aimed to develop population pharmacokinetic models for isoniazid specific to each regimen. The objectives were to estimate typical pharmacokinetic parameters and IIV, examine the influence of NAT2 phenotype and relevant clinical covariates, and evaluate model performance using standard diagnostics, posterior predictive checks, and visual predictive checks.

## 2. Materials and Methods

### 2.1. Study Design and Data

Participants were recruited in Sichuan Province between July 2024 and June 2025. The data were obtained from a prospective observational study of individuals with latent tuberculosis infection (LTBI) receiving tuberculosis preventive therapy.

Eligible participants had received at least one dose of a predefined regimen, had at least one evaluable plasma isoniazid (INH) concentration measurement, and provided written informed consent. Participants were excluded if dosing or sampling times could not be reliably determined, if key covariates (including NAT2 phenotype) were missing, or if concentration records were inconsistent with documented dosing histories.

Blood samples were collected after two weeks of treatment at predose and 1 h, 2 h, 4 h, 6 h, 8 h post-dose. Demographic, clinical, dosing, and sampling data were recorded using standardized case report forms and verified against source documents prior to inclusion in the population pharmacokinetic analysis dataset.

Pharmacokinetic data were derived from participants who received isoniazid (INH) as part of routine tuberculosis preventive treatment or study-directed treatment regimens. The analysis included individuals treated with one of three predefined regimens: 6 months of daily INH (6H), 3 months of daily INH plus rifampicin (3HR), or 3 months of twice-weekly INH plus rifapentine (3H2P2). Participants were eligible for inclusion if they had received at least one administered dose of the study regimen and contributed evaluable plasma concentration data for INH; concentration data for rifampicin or rifapentine were additionally included when these agents were co-administered.

This analysis was based on pooled pharmacokinetic observations collected from the enrolled study population according to protocol-defined or regimen-specific sampling schedules. For each participant, dosing records and blood sampling times were documented in detail to support reconstruction of individual exposure histories. In addition to concentration–time data, relevant demographic and clinical variables were recorded, including those considered biologically plausible or clinically relevant for pharmacokinetic variability assessment. These variables comprised, where available, age, sex, body weight, treatment regimen, dose level, and other baseline or treatment-related covariates collected in the source dataset.

Plasma concentration–time data for INH and, where applicable, rifampicin or rifapentine were assembled into a population pharmacokinetic analysis dataset. The dataset included participant identifiers, treatment regimen, nominal and actual dose administration records, sampling times, analyte concentrations, and prespecified covariates. Data were reviewed for internal consistency before analysis, including verification of chronology between dosing and sampling records and assessment of missing or implausible entries.

Concentrations below the lower limit of quantification (BLQ) were retained in the analysis dataset and handled as left-censored observations using the M3 likelihood-based method [12]. For each BLQ observation, the likelihood contribution was calculated as the probability that the true concentration at the corresponding sampling time was below the assay lower limit of quantification. This approach allowed for incorporation of all available information while avoiding bias associated with simple exclusion or imputation of BLQ observations.

### 2.2. Population Pharmacokinetic Modeling

Population pharmacokinetic analyses were performed separately for each regimen using a Bayesian nonlinear mixed-effects modeling framework. Structural models were defined for INH alone (6H) or jointly for INH and rifampicin (3HR) or rifapentine (3H2P2). Model parameters were estimated on the logarithmic scale to ensure parameter positivity, with natural-scale summaries reported for interpretability.

IIV was modeled using log-normal random effects on selected pharmacokinetic parameters. Residual unexplained variability was described using a proportional error model on the log scale. Weakly informative prior distributions were assigned to all fixed-effect parameters, variance components, and residual error terms. Priors were specified based on pharmacokinetic plausibility and previous population pharmacokinetic studies of isoniazid and related compounds, with sufficiently wide distributions to allow the observed data to dominate posterior inference.

Regimen-specific models were selected a priori because the three regimens differed in co-administered rifamycin, dosing frequency, sampling structure, and proportion of BLQ observations. The 6H regimen included INH alone, whereas the 3HR and 3H2P2 regimens required joint characterization of INH with rifampicin or rifapentine, respectively. In addition, sparse sampling and limited early absorption-phase information reduced the identifiability of shared absorption parameters across regimens.

A pooled unified model using a common INH structural model with regimen-specific covariate effects for rifamycin co-administration was also explored conceptually. Although the same one-compartment structure with first-order absorption and elimination could describe the overall concentration–time profiles, the unified model showed greater posterior uncertainty for absorption-related parameters and did not improve predictive performance compared with regimen-specific models. Therefore, separate regimen-specific models were retained to improve parameter identifiability and to allow for clearer interpretation of regimen-dependent NAT2 and rifamycin effects.

### 2.3. Covariate Analysis

Covariate effects were evaluated within each regimen using a prespecified set of covariates. The evaluated covariates included NAT2 acetylator phenotype, age, sex, body weight, treatment regimen, dose level, and clinically relevant factors such as food intake and diabetes status. NAT2 genotype was determined using PCR-based assays, and acetylator phenotype was assigned according to the number of reduced-function alleles: participants with two reduced-function alleles were classified as slow acetylators, those with one reduced-function allele as intermediate acetylators, and those with no reduced-function alleles as rapid acetylators. Covariates were incorporated multiplicatively on the natural scale through linear effects on log-transformed parameters. Covariate selection followed a stepwise procedure within each regimen. Candidate covariates were first evaluated individually in a single-covariate (univariate) analysis. Covariates identified in the screening step were subsequently assessed in a multivariable model. Covariate inclusion was guided by predefined criteria, including: (1) a clinically meaningful posterior median effect; (2) improved model predictive performance based on posterior predictive checks; and (3) reduction in unexplained IIV in the associated parameter. Covariate selection was additionally informed by biological plausibility and consistency across regimens. Retained covariate effects are reported as linear coefficients (β) and multiplicative effects (exp[β]). Combined covariate effects across regimens were calculated as weighted averages of regimen-specific regression coefficients, with weights based on the number of subjects in each regimen. For multiplicative covariates, exp(β) values were summarized using the same weighting approach.

### 2.4. Model Estimation and Uncertainty

Posterior distributions of model parameters were obtained using Markov chain Monte Carlo sampling. Convergence was assessed using standard diagnostic criteria, including visual inspection of trace plots and effective sample sizes. Parameter estimates are summarized as posterior means with 94% highest density intervals (HDIs). Relative standard error (RSE) was calculated as the ratio of posterior standard deviation to the absolute posterior mean.

### 2.5. Model Evaluation

Model performance was evaluated using a combination of graphical and simulation-based diagnostics. Goodness-of-fit plots included observed vs. posterior mean predicted concentrations and log-scale residuals vs. time. Posterior predictive checks (PPCs) compared observed and simulated concentration distributions and time-binned summary statistics. Visual predictive checks (VPCs) were performed by simulating concentration–time profiles using the final population pharmacokinetic model. The 5th, 50th, and 95th percentiles of simulated concentrations were summarized over time and compared with the corresponding observed percentiles. Observed concentrations were overlaid on the prediction intervals. For BLQ data, a BLQ VPC was generated by comparing the observed percentage of BLQ samples with the model-predicted percentage over time. Diagnostic plots were generated separately for each regimen.

VPCs for the rifamycin population pharmacokinetic models were performed and are provided in Supplementary Figure S1. Observed rifampicin/rifapentine concentrations were overlaid on the simulated prediction intervals to evaluate whether the models adequately described the central tendency and variability of rifamycin exposure.

### 2.6. Simulation and Exposure Analysis

Model-based simulations were performed using the final population pharmacokinetic model to evaluate drug exposure under different treatment regimens. Simulations were conducted in a virtual population reflecting the characteristics of the study cohort, including body weight distribution and NAT2 acetylator phenotype proportions.

Individual concentration–time profiles were generated based on the posterior parameter estimates. The maximum concentration (Cmax) was defined as the peak concentration over the dosing interval, and the area under the concentration–time curve (AUC) was calculated using numerical integration of the simulated profiles.

The magnitude of drug–drug interactions (DDIs) was quantified by comparing exposure metrics (Cmax and AUC) between regimens, expressed as geometric mean ratios.

### 2.7. Software

Bayesian nonlinear mixed-effects models were implemented in Stan through CmdStanR (version 0.9.0) in R version 4.2.3. Data import and management were performed using readr (version 2.1.5), dplyr (version 1.1.4), and tidyr (version 1.3.1); posterior draws were processed using the posterior package (version 1.6.1); and figures were generated using ggplot2 (version 3.5.1). Posterior inference was performed using Hamiltonian Monte Carlo with the No-U-Turn Sampler implemented in CmdStan (version 2.35.0). For each of the three independent regimen-specific models, four Markov chains were run with 2000 warm-up iterations and 2000 post-warm-up iterations per chain. The target acceptance probability was set to 0.95 and the maximum tree depth was set to 12.

Convergence was assessed using visual inspection of trace plots, the potential scale reduction factor R, effective sample size, and divergent transition diagnostics. Model parameters were estimated on the log scale to ensure positivity and summarized on the natural scale as posterior means with 94% HDIs.

Weakly informative priors were assigned to all fixed-effect parameters, IIV terms, residual error parameters, and covariate effects. Priors were chosen to reflect pharmacokinetic plausibility while remaining sufficiently broad to allow the observed data to dominate posterior inference. The full prior specification is provided in Supplementary Table S1.

## 3. Results

### 3.1. Study Population and Data

The population pharmacokinetic analysis included regimen-specific concentration–time data for isoniazid (INH) and, where applicable, rifampicin (in 3HR) or rifapentine (in 3H2P2). The 6H regimen comprised 62 subjects contributing 372 observations, the 3HR regimen comprised 53 subjects contributing 318 observations, and the 3H2P2 regimen comprised 58 subjects contributing 348 observations (Table 1). BLQ observations accounted for 25.3%, 29.9%, and 30.5% of the data for the 6H, 3HR, and 3H2P2 regimens, respectively (Table 1). INH dosing was fixed at 300 mg per dose across all three regimens, with weight-based rifampicin and rifapentine dosing in the combination regimens summarized in Table 1.

### 3.2. Population Parameter Estimates

Across all three regimens, INH concentration–time data were adequately described by a one-compartment model with first-order absorption and first-order elimination. More complex structural models did not materially improve posterior predictive performance or parameter identifiability.

Across regimens, the estimated allometric exponents for body weight were 0.728 for apparent oral clearance (CL/F) (RSE, 23.90%; 94% HDI, 0.412–1.070) and 0.999 for apparent volume of distribution (V/F) (RSE, 21.95%; 94% HDI, 0.606–1.436). IIV was estimated at 23.00% CV for CL/F (RSE, 17.86%; 94% HDI, 15.84–29.51) and 7.98% CV for V/F (RSE, 75.68%; 94% HDI, 0.001–22.79). The proportional residual error on the log scale was 0.895 (RSE, 2.76%; 94% HDI, 0.850–0.941). The absorption rate constant showed wider uncertainty than the disposition parameters, as reflected by broader HDIs and higher RSE values.

#### 3.2.1. 6H Regimen

For the 6H regimen, typical INH pharmacokinetic parameters were a CL/F of 21.83 L/h (RSE, 8.18%; 94% HDI, 18.53–25.23), a V/F of 51.03 L (RSE, 9.32%; 94% HDI, 42.50–60.62), and an absorption rate constant (Ka) of 2.88 h^−1^ (RSE, 62.09%; 94% HDI, 1.17–7.36) (Table 2).

#### 3.2.2. 3HR Regimen

For the 3HR regimen, typical INH pharmacokinetic parameters were a CL/F of 26.32 L/h (RSE, 9.14%; 94% HDI, 22.14–30.92), a V/F of 45.91 L (RSE, 10.83%; 94% HDI, 36.75–55.45), and a Ka of 2.71 h^−1^ (RSE, 66.08%; 94% HDI, 1.06–6.70) (Table 2).

#### 3.2.3. 3H2P2 Regimen

For the 3H2P2 regimen, typical INH pharmacokinetic parameters were a CL/F of 25.98 L/h (RSE, 8.84%; 94% HDI, 22.02–30.71), a V/F of 50.24 L (RSE, 10.19%; 94% HDI, 41.33–60.23), and a Ka of 2.88 h^−1^ (RSE, 60.51%; 94% HDI, 1.19–7.10) (Table 2).

#### 3.2.4. Covariate Effects

**Figure 1:**
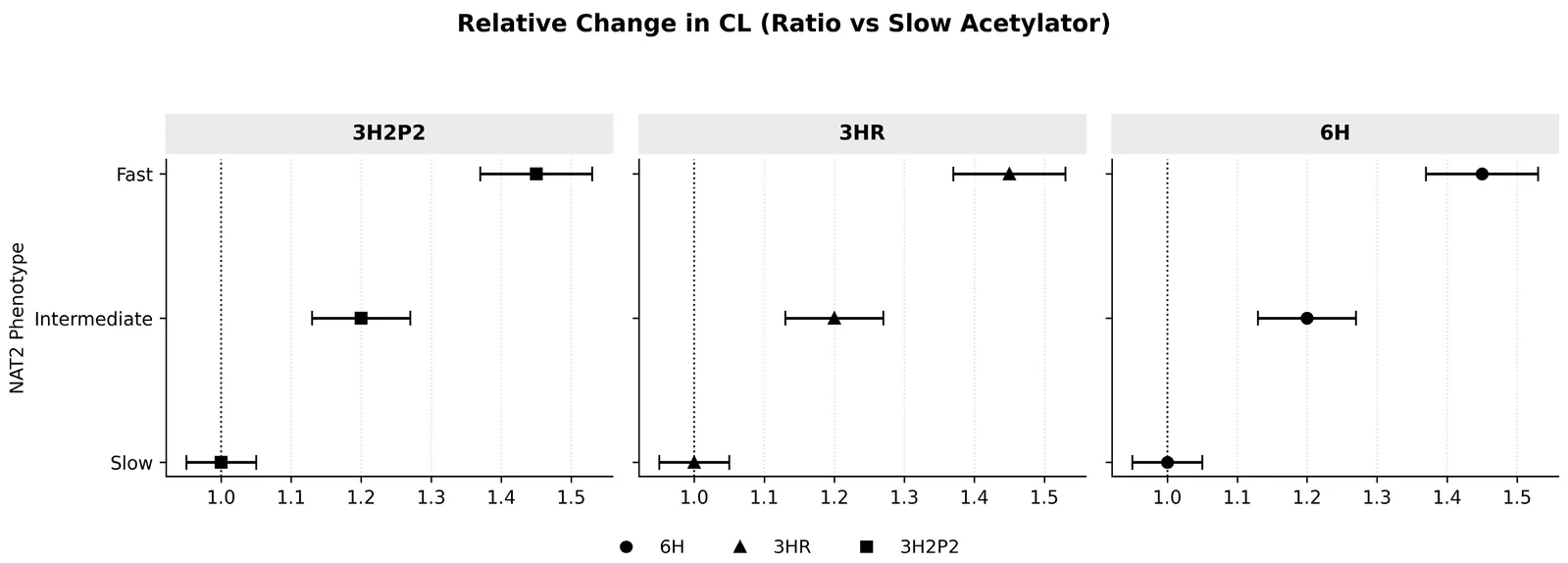
Effect of NAT2 acetylator phenotype on isoniazid (INH) clearance. Relative change in apparent oral clearance (CL/F) for intermediate and fast acetylators compared with the reference slow acetylator phenotype, presented as geometric mean ratio (94% highest density interval [HDI]) for the 6H, 3HR, and 3H2P2 regimens (left to right). INH, isoniazid; CL/F, apparent oral clearance; NAT2, N-acetyltransferase 2; HDI, highest density interval; 6H: 6 months of daily INH; 3HR: 3 months of daily INH plus rifampicin; 3H2P2: 3 months of twice-weekly INH plus rifapentine.

**Figure 2:**
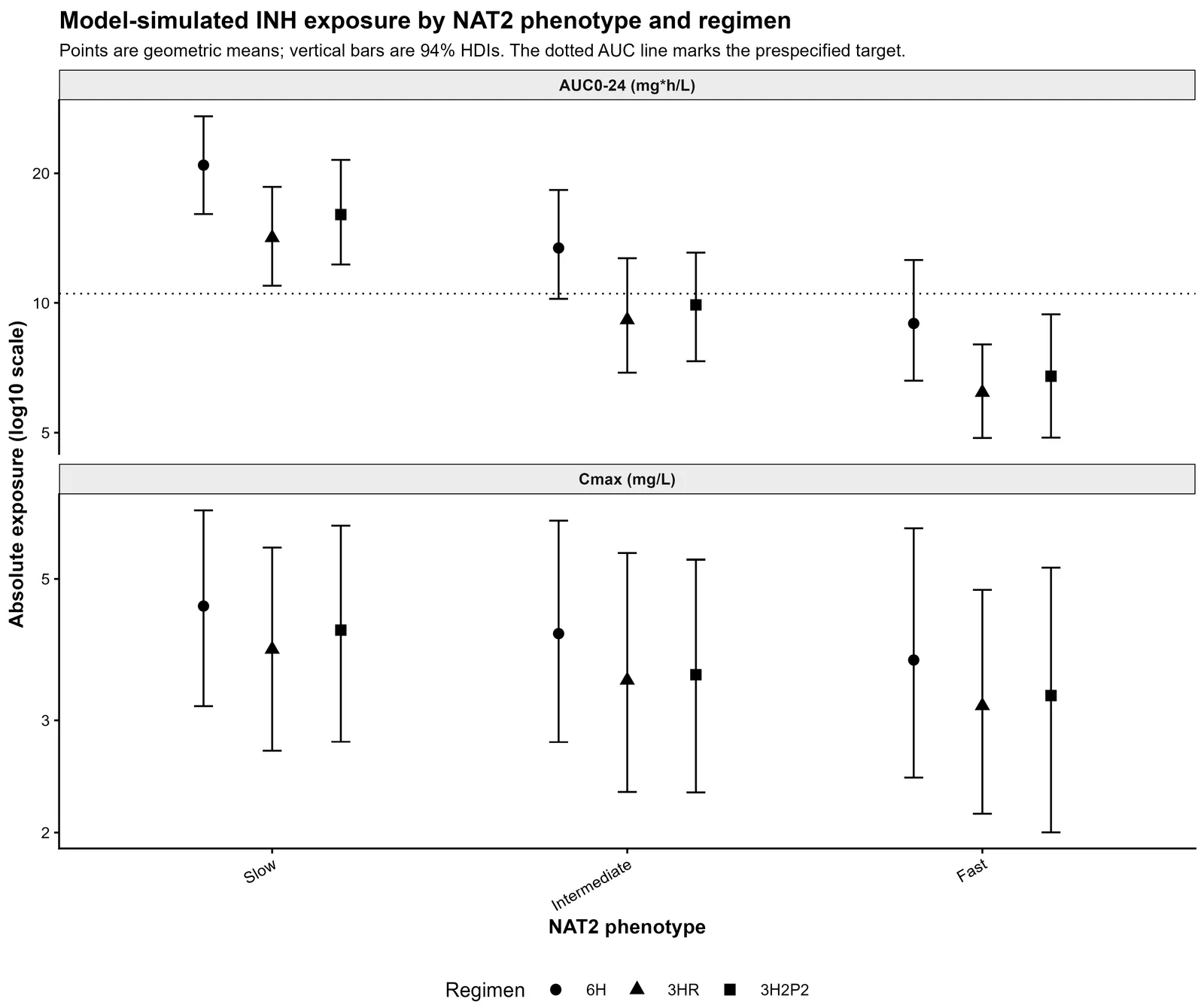
Model-simulated posterior predictive distributions of isoniazid exposure by treatment regimen and NAT2 acetylator phenotype. DDI assessment uses simulated exposure metrics, AUC_0–24_ and Cmax, rather than point concentration comparisons. AUC_0–24_ and Cmax are shown for 6H, 3HR, and 3H2P2 in fast, intermediate, and slow acetylators. Distributions are summarized using 94% highest density intervals (HDIs). The horizontal dotted line in the AUC_0–24_ panel indicates the prespecified efficacy-associated reference threshold of 10.52 mg·h/L, based on previously published data [13]. AUC_0–24_, area under the concentration-time curve from 0 to 24 h; Cmax, maximum plasma concentration; NAT2, N-acetyltransferase 2; HDI, highest density interval; 6H, 6 months of daily INH; 3HR, 3 months of daily INH plus rifampicin; 3H2P2, 3 months of twice-weekly INH plus rifapentine.

Retained covariate effects are summarized in Table 3 and illustrated in Figure 1 and Figure 2. Across all regimens, NAT2 acetylator status was consistently associated with INH clearance. Compared with the intermediate acetylator reference phenotype, NAT2 slow acetylators showed a 35.4% reduction in CL/F (effect ratio: 0.646; 94% HDI: 0.573–0.719), whereas fast acetylators exhibited a 48.4% increase in CL/F (effect ratio: 1.484; 94% HDI: 1.327–1.642). Body weight was also a significant covariate: a 10 kg increase in body weight was associated with an 11.3% (94% HDI: 6.3–17.1) increase in CL/F and a 15.9% (94% HDI: 9.2–23.5) increase in V/F. Post hoc CL/F estimates stratified by regimen and NAT2 phenotype are provided in Supplementary Table S2 and showed the same pattern, with fast acetylators having consistently higher CL/F than intermediate and slow acetylators.

#### 3.2.5. Drug–Drug Interaction Effects

The simulated isoniazid exposure profiles by regimen and NAT2 acetylator status are shown in Table 4. Across all regimens, NAT2 phenotype was the primary determinant of exposure, with AUC_0–24_ consistently lowest in fast acetylators and highest in slow acetylators. In the 6H regimen, the median AUC_0–24_ values were 8.81, 13.29, and 20.85 mg·h/L in fast, intermediate, and slow acetylators, respectively, and the corresponding probabilities of subtarget exposure were 84.5%, 4.1%, and 0%.

Both rifampicin (3HR) and rifapentine (3H2P2) further reduced INH exposure within each NAT2 subgroup. In fast acetylators, median AUC_0–24_ decreased to 6.20 mg·h/L with 3HR and 6.76 mg·h/L with 3H2P2, corresponding to AUC ratios of 0.69 and 0.75 relative to 6H, while the probability of subtarget exposure increased to 100% and 99%, respectively. Intermediate acetylators showed the same pattern, with probabilities of subtarget exposure increasing to 81.9% in 3HR and 65.7% in 3H2P2. In contrast, slow acetylators maintained relatively high exposure across regimens, and the probability of subtarget exposure remained low (0–1.2%).

Simulated Cmax values varied less markedly than AUC and remained broadly within the expected reference range across regimens. Overall, these findings suggest that rifamycin co-administration reduces INH exposure across NAT2 phenotypes, with the greatest impact in fast acetylators, who appear to be the subgroup at highest risk of subtarget exposure.

### 3.3. Model Evaluation

#### 3.3.1. Goodness-of-Fit and Residual Diagnostics

**Figure 3:**
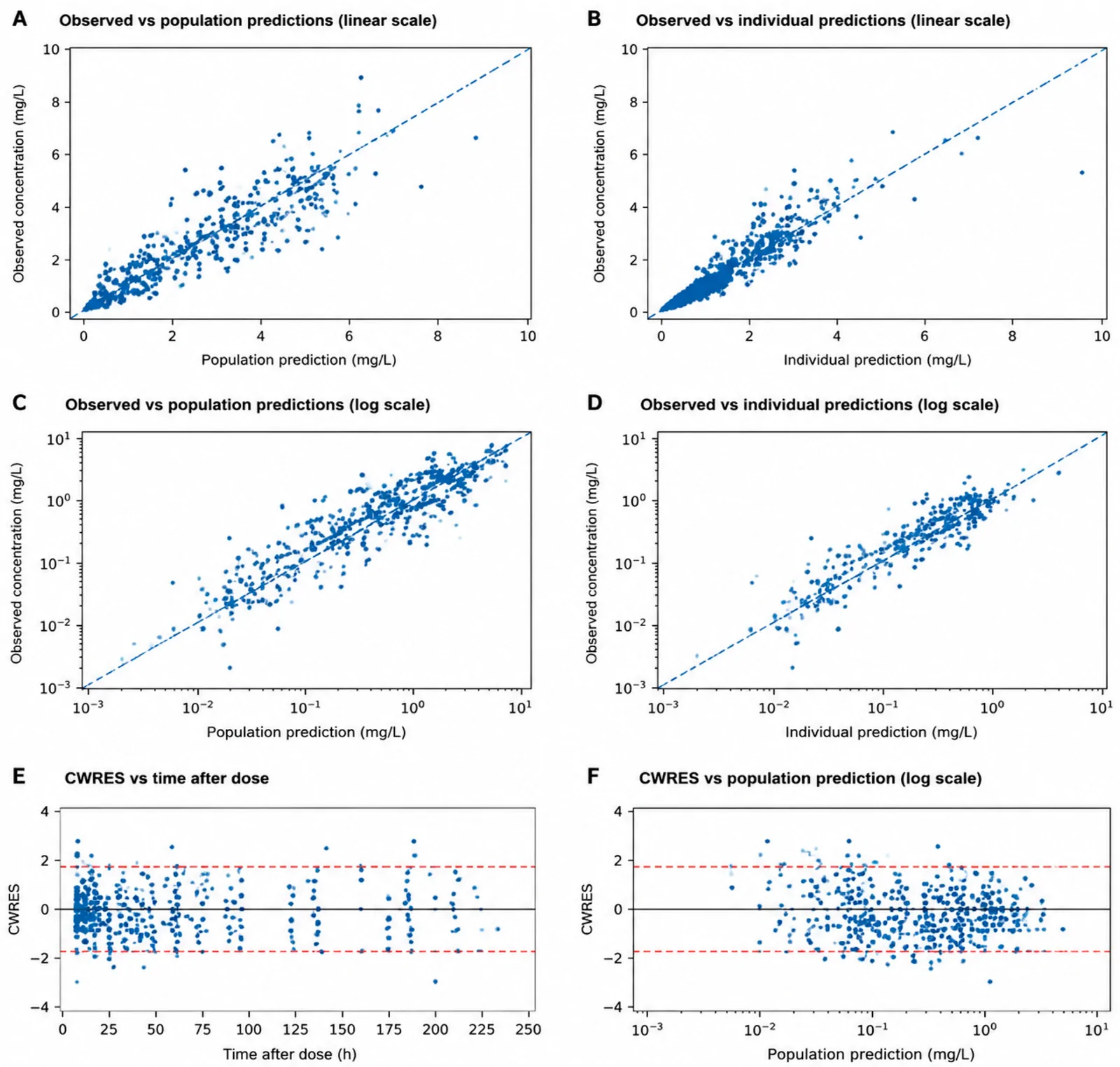
Goodness-of-fit diagnostics of the final population pharmacokinetic model. Observed concentrations vs. population predictions and individual predictions are presented on linear scales (**A**,**B**) and log-transformed scales (**C**,**D**). Conditional weighted residual (CWRES) vs. time after dose and population predictions are shown in panels (**E**,**F**), respectively. The dashed line represents the line of identity (y = x), and the red dashed lines indicate the ±1.96 reference limits for CWRES. No smoothing curves were applied.

Goodness-of-fit diagnostics supported the adequacy of the final models across the three regimens. Observed concentrations were generally consistent with posterior population and individual predictions, and no systematic deviation from the line of identity was observed. Conditional weighted residuals were centered around zero and showed no apparent trend over time or across predicted concentrations (Figure 3). Overall, no systematic bias was detected, supporting the selected structural and residual error models.

#### 3.3.2. Posterior Predictive and Visual Predictive Checks

**Figure 4:**
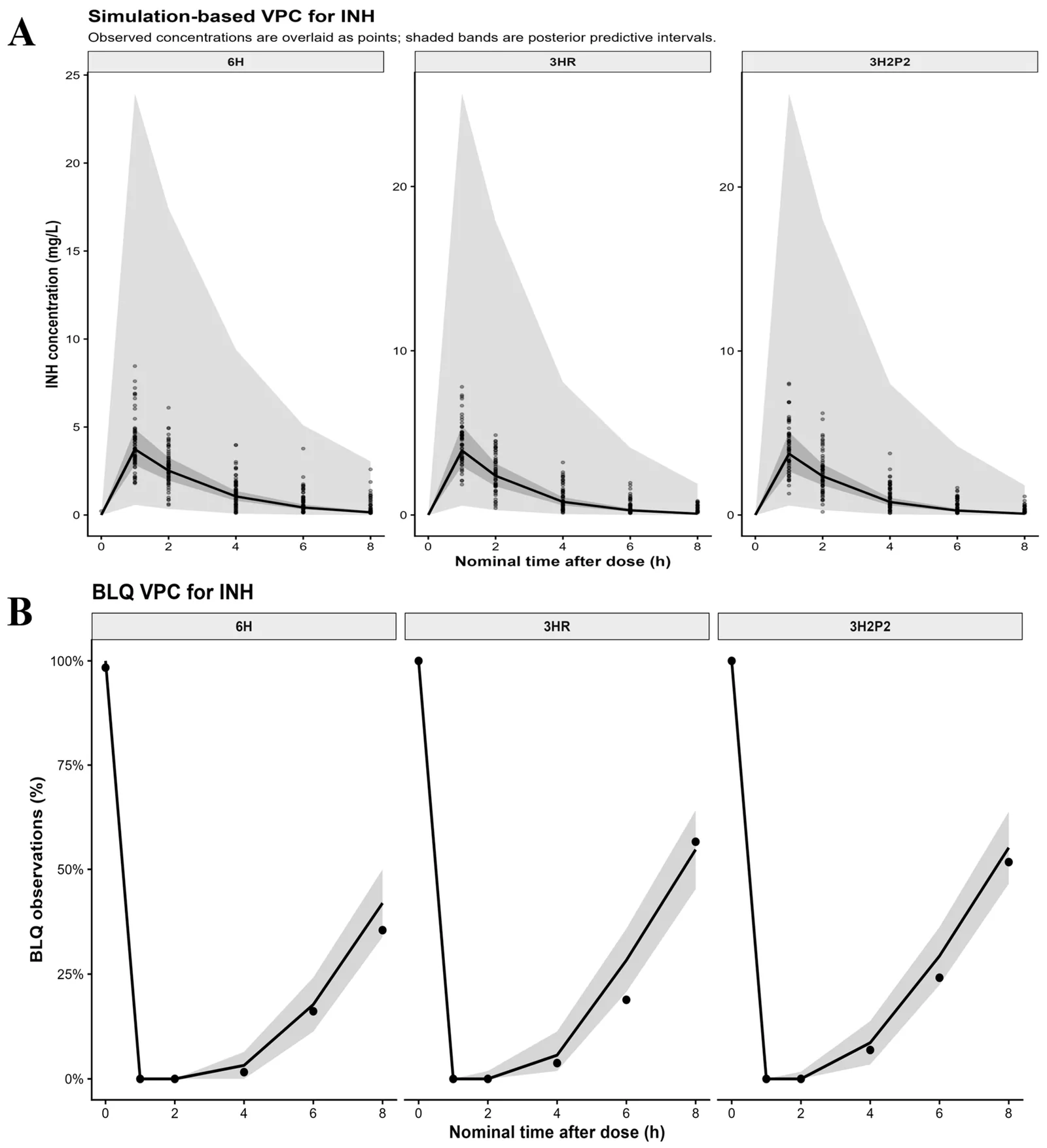
Visual predictive checks for isoniazid concentrations and below-quantification-limit observations stratified by treatment regimen. (**A**) Visual predictive check (VPC) of isoniazid (INH) concentrations for 6H, 3H2P2, and 3HR. Black points and lines show observed concentrations and median profiles; grey shaded areas indicate model-simulated 90% prediction intervals. (**B**) VPC of below-quantification-limit (BLQ) observations. Black points and lines show observed BLQ percentages, and grey shaded areas indicate simulated 90% prediction intervals. INH, isoniazid; BLQ, below the lower limit of quantification; 6H, daily isoniazid; 3H2P2, 3 months of twice-weekly INH plus rifapentine; 3HR, daily isoniazid plus rifampicin.

The posterior predictive performance of the final model was evaluated using prediction-corrected visual predictive checks (pcVPC), stratified by treatment regimen (6H, 3H2P2, and 3HR) and NAT2 acetylator phenotype (slow, intermediate, and fast) (Figure 4). Across all regimens and phenotype groups, the simulated median concentration–time profiles closely followed the observed medians throughout the dosing interval. The majority of observed median curves were centrally located within the corresponding simulated prediction intervals, indicating appropriate characterization of the central tendency of the data. Furthermore, the observed 5th and 95th percentiles were largely encompassed within the simulated 90% prediction intervals over time, with no consistent over- or under-prediction across regimens or phenotype strata. Although minor deviations were observed at early time points in certain subgroups, no systematic bias or time-dependent divergence was evident. Collectively, these pcVPC results demonstrate robust predictive performance of the final Bayesian population pharmacokinetic model across different treatment regimens and NAT2 phenotype categories.

## 4. Discussion

In this study, regimen-specific population pharmacokinetic models were developed to characterize the pharmacokinetics of INH administered as monotherapy (6H) or in combination with rifampicin (3HR) or rifapentine (3H2P2) for tuberculosis preventive therapy. The final models adequately described observed concentration–time profiles across regimens, as supported by diagnostic plots and predictive checks, indicating robust model performance and acceptable parameter identifiability. The inclusion of three widely used preventive regimens enabled a systematic evaluation of regimen-dependent differences in INH disposition and the relative contributions of genetic, physiological, and drug–drug interaction-related sources of variability. Although observations below the lower limit of quantification were common, particularly in the 6H regimen, these data were appropriately handled in the modeling framework and did not compromise overall model performance.

### 4.1. Regimen-Dependent Pharmacokinetics of Isoniazid

The one-compartment disposition model with first-order absorption and elimination for INH is consistent with previous population pharmacokinetic studies in both active tuberculosis and latent infection [7,14]. Evaluation of alternative structural models did not yield meaningful improvements in predictive performance or parameter precision. Notably, CL/F varied across regimens, with the lowest estimate observed for INH monotherapy (6H) and higher estimates observed when INH was co-administered with rifampicin (3HR) or rifapentine (3H2P2). This pattern is consistent with rifamycin-related induction of metabolic pathways affecting INH disposition [15,16]. By contrast, apparent V/F remained relatively similar across regimens, suggesting that regimen-dependent differences were mainly driven by clearance rather than distribution.

Absorption-related parameters were estimated with greater uncertainty, particularly in rifamycin-containing regimens. This likely reflects differences in co-administered drugs, dosing schedules, formulation-related factors, BLQ observations, and the limited number of samples collected during the early absorption phase. Although a pooled unified model was explored, it did not improve predictive performance and resulted in less stable estimation of absorption parameters. Therefore, regimen-specific models were retained to improve parameter identifiability and to enable clearer interpretation of regimen-dependent rifamycin effects under sparse-sampling conditions.

### 4.2. Influence of NAT2 Acetylator Status

NAT2 acetylator status was the most influential covariate affecting INH CL/F across all regimens [17,18]. Slow acetylators consistently exhibited reduced CL/F, whereas fast acetylators showed substantially increased CL/F relative to the reference phenotype. Notably, the magnitude of NAT2 effects was consistent across regimens, with slow acetylators demonstrating a 35% lower CL/F and fast acetylators a 50% higher CL/F, irrespective of rifamycin co-administration. Figure 4 demonstrates clear separation in exposure distributions across NAT2 phenotypes, with fast acetylators showing consistently lower AUC and Cmax, particularly under rifamycin co-administration, indicating a higher likelihood of subtherapeutic exposure. These findings confirm the dominant role of NAT2-mediated acetylation in INH disposition and demonstrate that pharmacogenetic variability remains a major driver of IIV even in preventive therapy settings [19]. Clinically, higher INH exposure in slow acetylators may increase the risk of concentration-dependent adverse effects, including hepatotoxicity and peripheral neuropathy, whereas fast acetylators—particularly when receiving rifampicin-containing regimens—may be at increased risk of subtherapeutic exposure [6,20,21].

### 4.3. Effects of Body Weight

Body weight was retained as a significant covariate influencing INH CL/F and V/F of distribution across regimens [22,23,24]. Inclusion of body weight on CL/F resulted in virtually no change in IIV in CL/F (from 22.90% CV to 23.00% CV), with a relative change of 0.01% (94% HDI: –39.5% to 44.4%), indicating that body size explained only a very limited proportion of the between-subject variability in INH disposition. Although modest differences in allometric exponents were observed between regimens, body weight consistently contributed to variability in INH exposure, supporting the continued use of weight-informed approaches in preventive therapy populations.

### 4.4. Drug–Drug Interactions with Rifamycin

This study provides a quantitative characterization of DDIs between INH and rifamycin in preventive therapy regimens. INH clearance was lowest during monotherapy in the 6H regimen and increased in the presence of rifampicin in the 3HR and 3H2P2 regimens, consistent with the known enzyme-inducing effects of rifampicin. The magnitude of the NAT2 acetylator effect on INH clearance observed in our study (slow acetylators: 35.4% lower; fast acetylators: 48.4% higher than the reference phenotype) is broadly in line with previous population pharmacokinetic studies of INH in both active and latent tuberculosis populations [20,25]. Direct comparisons of regimen-specific rifamycin effects are more limited, but the increases in clearance associated with rifampicin co-administration are comparable to those reported in earlier studies evaluating INH–rifampicin interactions, while rifapentine exhibited a smaller effect, as previously suggested by pharmacokinetic modeling studies of shorter preventive therapy regimens [26]. Rifampicin-containing regimens were associated with changes in systemic exposure, whereas no apparent differences in KA across regimens in exploratory analyses were supported in the final model.

### 4.5. Implications for Preventive Therapy Optimization

The analysis highlights the additive effects of NAT2 genotype and rifampicin-induced enzyme induction on INH CL/F. Fast acetylators receiving rifamycin-containing regimens represent a subgroup with particularly high CL/F and reduced exposure, whereas slow acetylators receiving INH monotherapy exhibit the highest exposures. These findings underscore that variability in INH pharmacokinetics during preventive therapy is driven by the combined influence of genetic and regimen-related factors. Although rifamycin-containing regimens reduced INH exposure by approximately 10–17%, slow acetylators without rifamycin co-administration exhibited the highest exposure, approaching but generally not exceeding reported toxicity thresholds. In contrast, fast acetylators receiving rifamycin-containing regimens showed the lowest exposure, with a greater likelihood of falling below efficacy-associated ranges. These findings indicate that the clinical relevance of DDI is dependent on NAT2 phenotype.

To aid clinical interpretation, these findings should be viewed in relation to published INH therapeutic drug monitoring reference values. Although preventive-therapy-specific exposure targets are not well established, prior studies have commonly used a Cmax reference range of 3–6 mg/L and an AUC_0–24h_ above 10.5 mg·h/L as an efficacy-associated benchmark, and an AUC_0–24h_ above 21.8 mg·h/L as a hepatotoxicity-risk margin [13]. Based on these reference values, slow acetylators receiving INH monotherapy may represent the subgroup with the greatest potential exposure-related safety concern, whereas fast acetylators receiving rifamycin-containing regimens may be most vulnerable to subtherapeutic exposure. These patterns are consistent with pharmacogenetic studies linking NAT2 slow acetylator status to higher INH exposure and hepatotoxicity, and NAT2 fast acetylator status to lower exposure and potentially less favorable efficacy outcomes [27,28]. Because our study did not include clinical endpoints or formal dose-optimization simulations, these findings should be interpreted as supporting targeted monitoring or future genotype-informed dosing studies rather than immediate dose-adjustment recommendations.

The results indicate that standard preventive therapy regimens do not yield comparable INH exposure across all individuals and regimens. While INH monotherapy may lead to relatively high exposure in slow acetylators, rifamycin-containing regimens—especially those including rifamycin—may result in reduced exposure in fast acetylators. These findings support consideration of regimen-specific and potentially genotype-informed strategies for optimizing INH preventive therapy. Although routine NAT2 genotyping is not currently standard practice, pharmacogenetic information may help identify individuals at increased risk of toxicity or subtherapeutic exposure.

### 4.6. Strengths and Limitations

The strengths of this study include evaluation of multiple preventive therapy regimens, integration of pharmacogenetic data, and rigorous model evaluation. This study has several limitations, including the absence of pharmacodynamic or clinical outcome data and uncertainty in absorption-related parameters due to sparse early sampling and BLQ observations. In addition, no independent external dataset was available for formal external validation; therefore, model evaluation was conducted using goodness-of-fit diagnostics, posterior predictive checks, visual predictive checks, and simulation-based assessment of exposure distributions.

## 5. Conclusions

In conclusion, this study provides a comprehensive, regimen-specific population pharmacokinetic characterization of isoniazid in tuberculosis preventive therapy. NAT2 acetylator status and rifamycin co-administration were identified as major, independent determinants of INH CL/F and exposure, with rifampicin exerting stronger interaction effects than rifapentine. These findings provide a quantitative basis for model-informed and potentially genotype-guided optimization of preventive therapy regimens to improve the efficacy and safety of tuberculosis prevention.

## Figures and Tables

**Table 1:** Summary of baseline characteristics and dosing information by regimen.

Variable	6H	3HR	3H2P2
Number of subjects	62	53	58
PK Observations	372	318	348
Nominal sampling times, h	0, 1, 2, 4, 6, 8	0, 1, 2, 4, 6, 8	0, 1, 2, 4, 6, 8
INH dose (mg/dose)	300	300	300
Rifampicin/Rifapentine dose (mg/dose)	/	450 (<50 kg) 600 (≥50 kg)	500 (<50 kg) 600 (≥50 kg)
Measured INH concentration median (mg/L), median (IQR)	1.7 (0.6, 3.1)	1.5 (0.6, 3.4)	1.5 (0.6, 3.0)
BLQ observations, *n* (%)	94 (25.3)	95 (29.9)	106 (30.5%)
Study location	Sichuan Province, China	Sichuan Province, China	Sichuan Province, China
Age (years), median (IQR)	38 (32.3, 45.3)	39 (33.3, 45.7)	38 (32.4, 46.5)
Male, *n* (%)	31 (50.0)	25 (47.2)	25 (43.1)
Weight (kg), median (IQR)	66.4 (59.0, 72.0)	61.7 (57.8, 69.1)	58.9 (53.9, 69.3)
NAT2 phenotype, *n* (%)			
Fast	24 (38.7%)	13 (24.5%)	19 (32.8%)
Intermediate	18 (29.0%)	21 (39.6%)	23 (39.6%)
Slow	20 (32.3%)	19 (35.9%)	16 (27.6%)

Baseline covariates, NAT2 phenotype distribution, observed concentration summary, and BLQ proportion are reported by regimen. Data are presented as *n* (%) or median (IQR), as appropriate. Percentages were calculated within each treatment regimen. INH dosing was fixed at 300 mg per dose in all three regimens. In the 3HR regimen, rifampicin was administered at 450 mg for participants weighing <50 kg and 600 mg for those weighing ≥50 kg. In the 3H2P2 regimen, rifapentine was administered at 500 mg for participants weighing <50 kg and 600 mg for those weighing ≥50 kg. BLQ observations were included in the concentration summaries and model dataset. Abbreviations: BLQ, below the lower limit of quantification; IQR, interquartile range; INH, isoniazid; NAT2, N-acetyltransferase 2; PK, pharmacokinetic; RIF, rifampicin; RPT, rifapentine; 6H, 6 months of daily isoniazid; 3HR, 3 months of daily isoniazid plus rifampicin; 3H2P2, 3 months of twice-weekly isoniazid plus rifapentine.

**Table 2:** Population Pharmacokinetic Parameter Estimates Across Regimens.

Parameter	Unit	Estimate (94% HDI)	RSE, %
Clearance (CL/F)			
6H	L/h	21.83 (18.53–25.23)	8.18
3HR	L/h	26.32 (22.14–30.92)	9.14
3H2P2	L/h	25.98 (22.02–30.71)	8.84
Volume of distribution (V/F)			
6H	L	51.03 (42.50–60.62)	9.32
3HR	L	45.91 (36.75–55.45)	10.83
3H2P2	L	50.24 (41.33–60.23)	10.19
Absorption rate constant (Ka)			
6H	1/h	2.88 (1.17–7.36)	62.09
3HR	1/h	2.71 (1.06–6.70)	66.08
3H2P2	1/h	2.88 (1.19–7.10)	60.51
Allometric exponents			
Weight on CL/F	-	0.728 (0.412–1.070)	23.90
Weight on V/F	-	0.999 (0.606–1.436)	21.95
Residual variability			
Proportional error (log-scale SD)	-	0.895 (0.850–0.941)	2.76
Inter-individual variability (IIV)			
IIV CL/F	%CV	23.00 (15.84–29.51)	17.86
IIV V/F	%CV	7.98 (0.001–22.79)	75.68

Parameter estimates are reported as posterior median, RSE%, and 94% HDI. Data are presented as posterior median (94% highest density interval [HDI]) and relative standard error (RSE, %). RSE values are reported to two decimal places. Regimen-specific CL/F, V/F, and Ka estimates represent typical population values for each treatment regimen. Inter-individual variability is reported as percent coefficient of variation (%CV). Abbreviations: CL/F, apparent oral clearance; V/F, apparent volume of distribution; Ka, absorption rate constant; IIV, inter-individual variability; RSE, relative standard error; HDI, highest density interval; %CV, percent coefficient of variation; 6H, 6 months of daily isoniazid; 3HR, 3 months of daily isoniazid plus rifampicin; 3H2P2, 3 months of twice-weekly isoniazid plus rifapentine.

**Table 3:** Summary of covariate effects on isoniazid pharmacokinetic parameters.

Covariate	Unit	Effect (94% HDI)
NAT2 acetylator status (vs. Intermediate)		
Slow	CL/F	–35.4% (–42.7 to –28.1)
Fast/Rapid	CL/F	+48.4% (+32.7 to +64.2)
Body weight (per 10 kg increase)	CL/F	+11.3% (+6.3 to +17.1)
Body weight (per 10 kg increase)	V/F	+15.9% (+9.2 to +23.5)

NAT2 effects are expressed relative to intermediate acetylator status and presented as percentage changes with 94% HDIs. Body-weight effects are presented per 10 kg increase. CL/F, apparent oral clearance; V/F, apparent volume of distribution; NAT2, N-acetyltransferase 2; HDI, highest density interval.

**Table 4:** Simulated isoniazid exposure by regimen and NAT2 acetylator status.

Regimen	NAT2	N	Effective Dose (mg)	AUC_0–24_ (mg·h/L), Median (94% HDI)	Cmax (mg/L), Median (94% HDI)	AUC Ratio (vs. 6H)	Cmax Ratio (vs. 6H)	P (Below Target) (%)
6H	Slow	20,000	300	20.85 (16.09–27.16)	4.52 (3.16–6.40)	1.00	1.00	0.0
6H	Intermediate	18,000	300	13.29 (10.22–18.30)	4.03 (2.77–6.16)	1.00	1.00	4.1
6H	Fast	24,000	300	8.81 (6.60–12.59)	3.64 (2.44–6.00)	1.00	1.00	84.5
3HR	Slow	19,000	240	14.10 (10.98–18.60)	3.83 (2.69–5.59)	0.68	0.86	1.2
3HR	Intermediate	21,000	240	8.99 (6.89–12.71)	3.39 (2.32–5.48)	0.68	0.85	81.9
3HR	Fast	13,000	240	6.20 (4.86–8.01)	3.13 (2.14–4.81)	0.69	0.85	100
3H2P2	Slow	16,000	255	16.04 (12.29–21.49)	4.13 (2.78–6.05)	0.77	0.92	0.4
3H2P2	Intermediate	23,000	255	9.85 (7.32–13.10)	3.50 (2.31–5.36)	0.74	0.86	65.7
3H2P2	Fast	19,000	255	6.76 (4.87–9.41)	3.25 (2.00–5.21)	0.75	0.88	99.0

Data are presented as median (94% HDI) unless otherwise specified. Exposure metrics were derived from simulations based on the final population pharmacokinetic model under standardized conditions, with continuous covariates, such as body weight, fixed to typical population values. This approach was used to isolate and directly compare the effects of regimen and NAT2 phenotype on INH exposure under controlled covariate conditions. AUC and Cmax ratios were calculated within each NAT2 phenotype relative to the corresponding subgroup in the 6H regimen (reference = 1). The probability of subtarget exposure was defined as P (AUC_0–24_ < 10.52 mg·h/L), where 10.52 mg·h/L was used as the efficacy-associated reference threshold [13]. Effective dose = nominal INH dose (300 mg) × relative bioavailability factor. Relative factor: 1.0 for 6H, 0.8 for 3HR, 0.85 for 3H2P2. 6H, 6 months of daily INH; 3HR, 3 months of daily INH plus rifampicin; 3H2P2, 3 months of twice-weekly INH plus rifapentine; AUC, area under the concentration–time curve (mg·h/L); Cmax, maximum plasma concentration (mg/L); HDI, highest density interval; NAT2, N-acetyltransferase 2.

## Data Availability

The original contributions presented in this study are included in the article/Supplementary Materials. Further inquiries can be directed to the corresponding authors.

## Supplementary Materials

The following supporting information can be downloaded at: https://www.mdpi.com/article/10.3390/pharmaceutics18080937/s1, Table S1: Prior distributions used in the Bayesian population pharmacokinetic model; Table S2: Exposure metrics and post-hoc CL/F estimates based on individual empirical Bayes estimates; Table S3: Simulation metadata for model-based exposure analysis; Table S4: Overall model-simulated isoniazid exposure metrics by treatment regimen; Table S5: Separation of model-simulated isoniazid exposure by NAT2 acetylator phenotype across treatment regimens; Table S6: Model-simulated isoniazid exposure by regimen and NAT2 phenotype: geometric means, 94% HDIs, and medians; Table S7: Effect of body weight on unexplained inter-individual variability in CL/F; Table S8: Sensitivity analysis excluding the potentially influential 6H observation; Figure S1: Visual predictive checks (VPCs) for the rifamycin population pharmacokinetic models. (A) Rifampicin population pharmacokinetic model. (B) Rifapentine population pharmacokinetic model. Observed concentrations are overlaid on the simulated prediction intervals to evaluate model adequacy and assess whether the models capture the central tendency and variability of rifamycin exposure across nominal sampling times; Figure S2: Distribution of model-simulated isoniazid (INH) exposure (AUC_0–24_, mg·h/L) by NAT2 acetylator phenotype and treatment regimen.Boxplots summarize the posterior predictive distributions of area under the concentration–time curve from 0 to 24 h (AUC_0–24_) for each regimen (6H, 3HR, 3H_2_P_2_) and NAT2 phenotype (slow, intermediate, fast). Simulations were performed using the final Bayesian population pharmacokinetic model, incorporating inter-individual variability and covariate effects. The lower and upper hinges of each box correspond to the 25th and 75th percentiles, with the median indicated by the central line. Whiskers extend to the most extreme data points within 1.5 times the interquartile range. Individual raw data points are not shown (jitter omitted) to avoid overplotting and improve clarity. This figure complements Table 4 and Supplementary Table S2 by visualizing the full distributional separation of INH exposure across phenotypes and regimens. Abbreviations: AUC_0–24_, area under the concentration–time curve from 0 to 24 h; INH, isoniazid; NAT2, N-acetyltransferase 2; 6H, 6 months of daily INH; 3HR, 3 months of daily INH plus rifampicin; 3H_2_P_2_, 3 months of twice-weekly INH plus rifapentine; Supplementary Code: Code used for the population pharmacokinetic modeling and model-based exposure analysis.

## Abbreviations

The following abbreviations are used in this manuscript:

INH	Isoniazid
TB	Tuberculosis
6H	6 Months of Daily INH
3HR	3 Months of Daily INH plus Rifampicin
3H2P2	3 Months of Twice-Weekly INH plus Rifapentine
NAT2	N-acetyltransferase 2
LTBI	Latent Tuberculosis Infection
BLQ	Below The Lower Limit of Quantification
HDIs	Highest Density Intervals
RSE	Relative Standard Error
PPCs	Posterior Predictive Checks
VPCs	Visual Predictive Checks
AUC	The Area Under The Concentration–Time Curve
Cmax	The Maximum Concentration
DDIs	Drug–Drug Interactions
RIF	Rifampicin
RPT	Rifapentine
PRED	Population Prediction
IPRED	Individual Prediction
CWRES	Conditional weighted residuals
pcVPC	Prediction-Corrected Visual Predictive Check
IIV	Inter-Individual Variability
CL/F	Apparent Oral Clearance
V/F	Volume of Distribution
Ka	Absorption Rate Constant
