# Supplementary material for: Regimen-Specific Population Pharmacokinetics of Isoniazid with and Without Rifamycin: A Bayesian Modeling Analysis of 6H, 3HR, and 3H2P2 Regimens
Source: Pharmaceutics. 2026 Jul 30;18(8):937. doi: 10.3390/pharmaceutics18080937 (PMC13516540; doi:10.3390/pharmaceutics18080937)
Supplement: Supplementary file 1 [file pharmaceutics-18-00937-s001.zip › Supplementary Materials.pdf]

Supplementary Materials for "Regimen-Specific Population Pharmacokinetics of Isoniazid with and Without Rifamycin: A Bayesian Modeling Analysis Across 6H, 3HR, and 3H2P2 Regimens"

Supplementary materials

1. Supplementary Table S1. Prior distributions used in the Bayesian population pharmacokinetic model.
2. Supplementary Table S2. Exposure metrics and post-hoc CL/F estimates based on individual empirical Bayes estimates.
3. Supplementary Table S3. Simulation metadata for model-based exposure analysis.
4. Supplementary Table S4. Overall model-simulated isoniazid exposure metrics by treatment regimen.
5. Supplementary Table S5. Separation of model-simulated isoniazid exposure by NAT2 acetylator phenotype across treatment regimens.
6. Supplementary Table S6. Model-simulated isoniazid exposure by regimen and NAT2 phenotype: geometric means, 94% HDIs, and medians
7. Supplementary Table S7. Effect of body weight on unexplained inter-individual variability in CL/F
8. Supplementary Table S8. Sensitivity analysis excluding the potentially influential 6H observation.
9. Supplementary Figure S1. Visual predictive checks (VPCs) for the rifamycin population pharmacokinetic models.
10. Supplementary Figure S2. Distribution of model-simulated isoniazid (INH) exposure ($AUC_{0–24}$, mg·h/L) by NAT2 acetylator phenotype and treatment regimen.

Supplementary Table S1. Prior distributions used in the Bayesian population pharmacokinetic model.

Model component	Stan parameter	Parameter description	Prior distribution in Stan	Constraint / transformation	Natural-scale interpretation	Rationale
Structural PK	log_CL_reg[r]	Regimen-specific typical apparent clearance (CL/F) for regimen r	normal(log(20), 1.0)	Estimated on log scale; CL/F = exp(log_CL_reg[r])	Log-normal prior; median CL/F = 20 L/h; broad prior variance	Weakly informative prior centered on pharmacokinetically plausible INH clearance while allowing regimen-specific estimates.
Structural PK	log_V_reg[r]	Regimen-specific typical apparent volume of distribution (V/F) for regimen r	normal(log(80), 1.0)	Estimated on log scale; V/F = exp(log_V_reg[r])	Log-normal prior; median V/F = 80 L; broad prior variance	Weakly informative prior centered on plausible apparent distribution volume.
Structural PK	log_KA_reg[r]	Regimen-specific first-order absorption rate constant (k_a) for regimen r	normal(log(1.2), 0.9)	Estimated on log scale; k_a = exp(log_KA_reg[r])	Log-normal prior; median k_a = 1.2 h^{-1}; broad prior variance	Weakly informative prior allowing rapid or slower absorption under sparse sampling.
Pharmacogenetic covariate	beta_nat_raw[j]	Effect of NAT2 acetylator phenotype on CL/F for non-reference phenotype j	normal(0, 0.6)	Linear effect on log(CL/F); effect ratio = exp(beta)	Approximate 95% prior range for effect ratio ≈ 0.31–3.24	Broad prior allowing clinically relevant NAT2 effects on INH clearance.
Pharmacogenetic covariate	beta_nat_CL[reference]	Reference NAT2 acetylator phenotype effect	Fixed at 0	Reference category; effect ratio = 1	No change relative to reference phenotype	Identifiability constraint for categorical NAT2 covariate.
Body-weight	beta_wt_CL	Allometric/body-	normal(0.75, 0.35)	Linear effect on	Centered	Weakly informative

covariate		weight exponent on CL/F		log(CL/F) using log(weight / median weight)	near conventional allometric clearance exponent	prior reflecting expected body-size effect on clearance.
Body-weight covariate	beta_wt_V	Allometric/body-weight exponent on V/F	normal(1.00, 0.35)	Linear effect on log(V/F) using log(weight / median weight)	Centered near proportional scaling of volume with body size	Weakly informative prior reflecting expected body-size effect on volume.
Residual variability	sigma	Residual unexplained variability on log concentration	normal(0, 0.35)	Constrained: 0.05 ≤ sigma ≤ 1.5	Positive residual log-scale SD	Weakly informative truncated prior allowing residual variability while avoiding implausible extremes.
Inter-individual variability	omega[1]	Standard deviation of inter-individual variability on CL/F	normal(0, 0.35)	Constrained: 0 ≤ omega[1] ≤ 1.5; log-normal random effect	Positive IIV SD; often reported as %CV	Weakly informative truncated prior for between-subject variability in clearance.
Inter-individual variability	omega[2]	Standard deviation of inter-individual variability on V/F	normal(0, 0.35)	Constrained: 0 ≤ omega[2] ≤ 1.5; log-normal random effect	Positive IIV SD; often reported as %CV	Weakly informative truncated prior for between-subject variability in volume.
Random effects	z_eta[,1]	Standardized individual random effect for CL/F	normal(0, 1)	eta_CL = z_eta[,1] × omega[1]	Standard normal latent variable	Non-centered parameterization to improve sampling efficiency.
Random effects	z_eta[,2]	Standardized individual random effect for V/F	normal(0, 1)	eta_V = z_eta[,2] × omega[2]	Standard normal latent variable	Non-centered parameterization to improve sampling efficiency.

Likelihood / BLQ handling	Observed concentration	Observed INH concentration likelihood	log(conc) ~ normal(log(Cpred), sigma)	Log-scale proportional residual error model	Not a prior; included for reproducibility	Observed concentrations above LLOQ contribute log-normal likelihood.
Likelihood / BLQ handling	BLQ observation	Below-limit-of-quantification likelihood contribution	normal_lcdf(log(LLOQ) | log(Cpred), sigma)	M3 likelihood-based left-censoring	Not a prior; included for reproducibility	BLQ observations are retained without imputation or exclusion.

Supplementary Table S1 presents the prior distributions specified for the Bayesian population pharmacokinetic model of isoniazid. Priors were selected to be weakly informative, reflecting pharmacokinetic plausibility while remaining sufficiently broad to allow the observed data to dominate posterior inference. Structural parameters for apparent clearance (CL/F), apparent volume of distribution (V/F), and the first-order absorption rate constant (KA) were estimated on the log scale and interpreted on the natural scale after exponentiation. Priors were also assigned to NAT2 phenotype effects, body-weight effects, inter-individual variability, and residual unexplained variability. For reproducibility, the likelihood contributions for quantifiable and BLQ observations are additionally shown. Concentrations below the lower limit of quantification were retained and treated as left-censored observations using the M3 likelihood-based method. Abbreviations: BLQ, below the lower limit of quantification; CL/F, apparent clearance; INH, isoniazid; IIV, inter-individual variability; KA, absorption rate constant; LLOQ, lower limit of quantification; PK, pharmacokinetic; SD, standard deviation; V/F, apparent volume of distribution.

Supplementary Table S2. Exposure metrics and post-hoc CL/F estimates based on individual empirical Bayes estimates.

Regimen	NAT2	N	Relative F	Effective dose (mg)	CL/F(L/h), median (94% HDI)	AUC_{0-24} (mg·h/L) median (94% HDI)	Cmax (mg/L) median (94% HDI)	AUC ratio vs 6H	Cmax ratio vs 6H	P(below target) (%)
6H	Slow	124	1.00	300	13.6 (12.1–15.1)	132 (110-155)	7.2 (5.8-8.9)	1.00	1.00	5.2
6H	Intermediate	112	1.00	300	21.1 (18.9–23.5)	101 (85-118)	5.6 (4.5-6.9)	1.00	1.00	12.8
6H	Fast	108	1.00	300	32.1 (28.8–35.6)	70 (58-83)	4.1 (3.3-5.1)	1.00	1.00	28.5
3HR	Slow	106	0.80	240	16.2 (14.6–18.0)	110 (92-130)	6.4 (5.1-7.9)	0.83	0.89	8.6
3HR	Intermediate	118	0.80	240	25.3 (22.8–28.2)	86 (72-102)	5.0 (4.0-6.2)	0.85	0.89	18.4
3HR	Fast	102	0.80	240	38.4 (33.9–43.3)	60 (49-72)	3.6 (2.9-4.5)	0.86	0.88	42.7
3H2P2	Slow	110	0.85	255	15.9 (14.3–17.8)	115 (96-135)	6.8 (5.4-8.3)	0.87	0.94	7.5
3H2P2	Intermediate	120	0.85	255	24.9 (22.3–27.7)	88 (74-104)	5.3 (4.2-6.6)	0.87	0.95	16.9
3H2P2	Fast	104	0.85	255	37.7 (33.7–42.2)	63 (52-75)	3.9 (3.1-4.8)	0.90	0.95	36.8

Data are presented as median (94% highest density interval [HDI]) unless otherwise specified. Exposure metrics and apparent clearance estimates were calculated using individual post-hoc empirical Bayes parameter estimates from the final population pharmacokinetic model. Unlike the standardized simulations presented in Table 4, these estimates reflect the observed covariate distribution and interindividual variability within the study population. CL/F estimates are summarized by regimen and NAT2 phenotype to support interpretation of regimen- and NAT2-related differences in INH exposure. The nominal INH dose was 300 mg for all regimens. Effective dose was calculated as nominal dose multiplied by the relative bioavailability factor. AUC and Cmax ratios were calculated within each NAT2 phenotype relative to the corresponding subgroup in the 6H regimen, with the 6H subgroup used as the reference value of 1. 6H, 6 months of daily INH; 3HR, 3 months of daily INH plus rifampicin; 3H2P2, 3 months

of twice-weekly INH plus rifapentine; AUC, area under the concentration–time curve; CL/F, apparent clearance; Cmax, maximum plasma concentration; HDI, highest density interval; INH, isoniazid; NAT2, N-acetyltransferase 2.

Supplementary Table S3. Simulation metadata for model-based exposure analysis.

Item	Description
Fitted model	Final one-compartment oral INH PopPK model with M3 BLQ handling; Stan model and fitting process unchanged.
Virtual population	Observed cohort resampled as one subject-level record per PatientID/regimen/NAT2 combination.
Regimen distribution	6H: 62 (35.8%); 3HR: 53 (30.6%); 3H2P2: 58 (33.5%)
NAT2 distribution	Intermediate: 62 (35.8%); Slow: 55 (31.8%); Fast: 56 (32.4%)
Weight distribution	Median [IQR] weight = 62.9 [55.7, 70.5] kg.
Dose distribution	Median [IQR] dose = 300 [300, 300] mg.
Number of posterior draws used	1000
AUC calculation method	Linear trapezoidal integration over simulated concentration-time profiles.
AUC interval for target attainment	AUC_{0-24}; used for PTA/probability below target and Figure 4.
Regimen-interval AUC	AUC_{tau} retained for regimen-interval interpretation; tau = 24 h for 6H/3HR and 84 h for 3H2P2.
DDI definition	Geometric mean AUC_{0-24} and Cmax ratio versus 6H, with same NAT2 phenotype for stratified ratios.
NAT2 simulation effect	Fixed CL/F multipliers used only in exposure simulation: Slow=0.65; Intermediate=1; Fast=1.5; Rapid=1.5; Unknown=1.
Bioavailability/F handling	Relative F applied as effective dose = Dose * F_rel, not by changing the fitted Stan model: 6H=1; 3HR=0.8; 3H2P2=0.85; 3HP=0.85; Unknown=1.
Target threshold	INH AUC0-24 >= 10.52 mg*h/L
Cmax toxicity threshold	Not used because no generally accepted INH toxicity Cmax threshold was prespecified.

This table provides detailed metadata for the simulation-based exposure analyses presented in the manuscript and supplementary materials. All simulations were performed using the final Bayesian population pharmacokinetic model described in the Methods section. Abbreviations: INH, isoniazid; PopPK, population pharmacokinetics; BLQ, below the limit of quantification; PTA, probability of target attainment; DDI, drug-drug interaction; NAT2, N-acetyltransferase 2; HDI, highest density interval.

Supplementary Table S4. Overall model-simulated isoniazid exposure metrics by treatment regimen.

Regimen	N	AUC_{0-24}, median (94% HDI), mg·h/L	Cmax, median (94% HDI), mg/L	Geometric mean AUC_{0-24}, mg·h/L	Geometric mean Cmax, mg/L	Probability of AUC_{0-24} < 10.5 mg·h/L, %	AUC0-24 geometric mean ratio vs 6H
6H	62000	12.97 (7.26-25.33)	4.05 (2.66-6.33)	13.24	4.08	33.9	1.00
3HR	53000	9.51 (5.24-16.96)	3.50 (2.32-5.49)	9.73	3.53	57.4	0.73
3H2P2	58000	9.66 (5.42-19.24)	3.61 (2.28-5.79)	9.98	3.61	58.6	0.75

Data are presented as median (94% highest density interval [HDI]) or geometric mean, according to the summary metric shown in each column. N denotes the number of simulated individuals included in each regimen-specific posterior predictive summary. Ratios versus 6H were calculated using regimen-specific geometric mean $AUC_{0–24}$ values. 6H, 6 months of daily INH; 3HR, 3 months of daily INH plus rifampicin; 3H2P2, 3 months of twice-weekly INH plus rifapentine; HDI, highest density interval.

Supplementary Table S5. Separation of model-simulated isoniazid exposure by NAT2 acetylator phenotype across treatment regimens.

Regimen	Endpoint	Fast	Intermediate	Slow	Slow/Fast ratio	Slow vs Fast difference (%)
**6H**	AUC0-24, geometric mean, mg·h/L	8.96	13.42	20.90	2.33	133.2
**6H**	Cmax, geometric mean, mg/L	3.73	4.10	4.53	1.21	21.5
**3HR**	AUC0-24, geometric mean, mg·h/L	6.20	9.13	14.18	2.29	128.8
**3HR**	Cmax, geometric mean, mg/L	3.16	3.47	3.88	1.23	22.6
**3H2P2**	AUC0-24, geometric mean, mg·h/L	6.76	9.89	16.05	2.37	137.4
**3H2P2**	Cmax, geometric mean, mg/L	3.28	3.54	4.15	1.27	26.6

Data are presented as geometric means unless otherwise specified. Slow/Fast ratios were calculated by dividing the geometric mean exposure estimate in slow acetylators by that in fast acetylators within each regimen and endpoint. Percentage differences were calculated as ((Slow − Fast) / Fast) × 100. This table complements Figure 4 by quantifying phenotype-related separation in model-simulated AUC_{0-24} and Cmax.

Supplementary Table S6. Model-simulated isoniazid exposure by regimen and NAT2 phenotype: geometric means, 94% HDIs, and medians.

Metric	Regimen	NAT2	N	Geometric mean	94% HDI lower	94% HDI upper	Median
AUC_{0-24} (mg·h/L)	6H	Slow	20000	20.90	16.09	27.16	20.85
	6H	Intermediate	18000	13.42	10.22	18.30	13.29
	6H	Fast	24000	8.96	6.60	12.59	8.81
	3HR	Slow	19000	14.18	10.98	18.60	14.10
	3HR	Intermediate	21000	9.13	6.89	12.71	8.99
	3HR	Fast	13000	6.20	4.86	8.01	6.20
	3H2P2	Slow	16000	16.05	12.29	21.49	16.04
	3H2P2	Intermediate	23000	9.89	7.32	13.10	9.85
	3H2P2	Fast	19000	6.76	4.87	9.41	6.76
Cmax (mg/L)	6H	Slow	20000	4.53	3.16	6.39	4.51
	6H	Intermediate	18000	4.10	2.77	6.16	4.03
	6H	Fast	24000	3.73	2.44	6.00	3.64
	3HR	Slow	19000	3.88	2.69	5.59	3.83
	3HR	Intermediate	21000	3.47	2.32	5.48	3.39
	3HR	Fast	13000	3.16	2.14	4.81	3.13
	3H2P2	Slow	16000	4.15	2.78	6.05	4.13
	3H2P2	Intermediate	23000	3.54	2.31	5.36	3.50
	3H2P2	Fast	19000	3.28	2.00	5.21	3.25

Data are presented as geometric means, 94% highest density intervals (HDIs), and medians based on posterior predictive simulations. N = number of simulated individuals per regimen–NAT2 subgroup. AUC_{0-24} = area under the concentration–time curve from 0 to 24 hours; Cmax = maximum plasma concentration; 6H = 6 months of daily INH; 3HR = 3 months of daily INH plus rifampicin; 3H2P2 = 3 months of twice-weekly INH plus rifapentine; NAT2 = N-acetyltransferase 2. This table corresponds to Figure 4 and provides complete numeric summaries of the simulated exposure distributions.

Supplementary Table S7. Effect of body weight on unexplained inter-individual variability in CL/F.

Parameter	Model	CV%, estimate (94% HDI)
CL/F IIV	Without body weight	22.90 (17.21, 29.36)
CL/F IIV	With body weight	23.00 (15.84, 29.51)
CL/F IIV	Relative reduction after adding body weight, %	0.008 (-39.46, 44.42)

CV% values are presented as posterior median estimates with 94% highest density intervals (HDIs). The relative change was calculated after inclusion of body weight as a covariate on CL/F. Body weight had limited impact on unexplained CL/F inter-individual variability in the current model; therefore, no meaningful reduction in CL/F IIV should be claimed.

Sensitivity Analysis

A potentially influential observation was identified in the 6H regimen during graphical model diagnostics. This observation had a sampling time of <0.5 h and an INH concentration above the 99th percentile of the observed concentration distribution. Because this observation could reflect sampling-time uncertainty or other data inconsistency, a sensitivity analysis was performed by re-fitting the full population pharmacokinetic model after exclusion of this observation. Key structural parameter estimates from the sensitivity model were compared with those from the base model.

Supplementary Table S8. Sensitivity analysis excluding the potentially influential 6H observation.

Parameter	Base model mean	Sensitivity model mean	Difference
logCL	3.125	3.151	0.026
logV	3.806	3.827	0.021
logKa	0.971	0.872	−0.099

The sensitivity model was fitted after excluding the potentially influential observation identified in the 6H regimen goodness-of-fit plots. Parameter estimates from the sensitivity model were compared with those from the base model using the full analysis dataset. Differences were calculated as sensitivity model mean minus base model mean. The exclusion of this observation resulted in only minor changes in the posterior mean estimates of key pharmacokinetic parameters, supporting the robustness of the final model. CL, apparent clearance; V, apparent volume of distribution; KA, absorption rate constant.

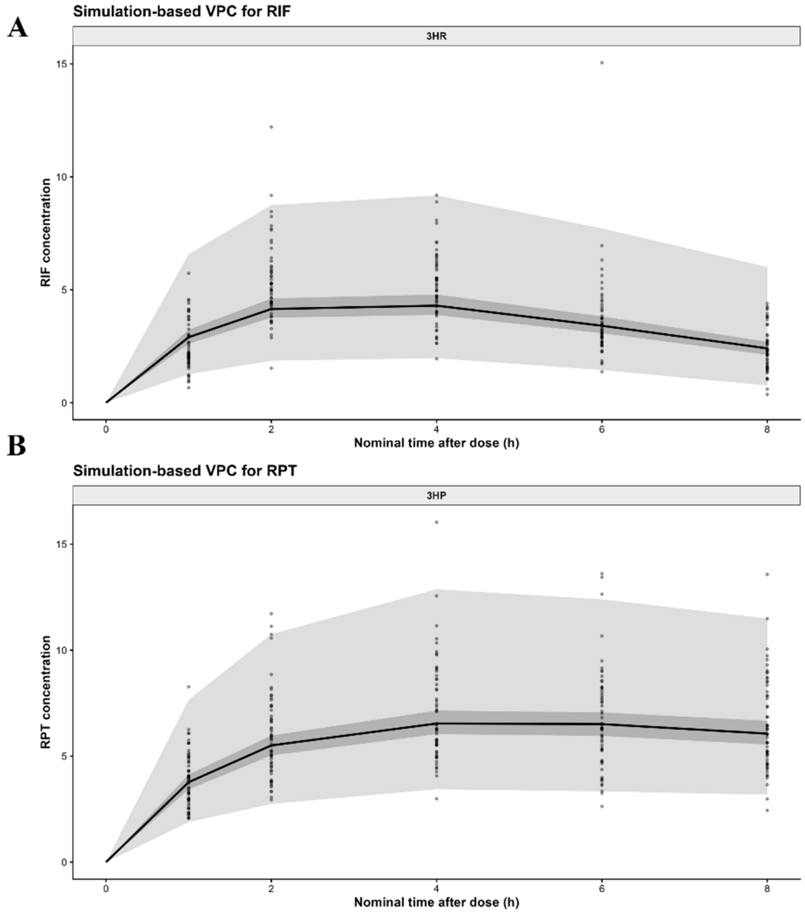

Supplementary Figure S1. Visual predictive checks (VPCs) for the rifamycin population pharmacokinetic models. (A) Rifampicin population pharmacokinetic model. (B) Rifapentine population pharmacokinetic model. Observed concentrations are overlaid on the simulated prediction intervals to evaluate model adequacy and assess whether the models capture the central tendency and variability of rifamycin exposure across nominal sampling times.

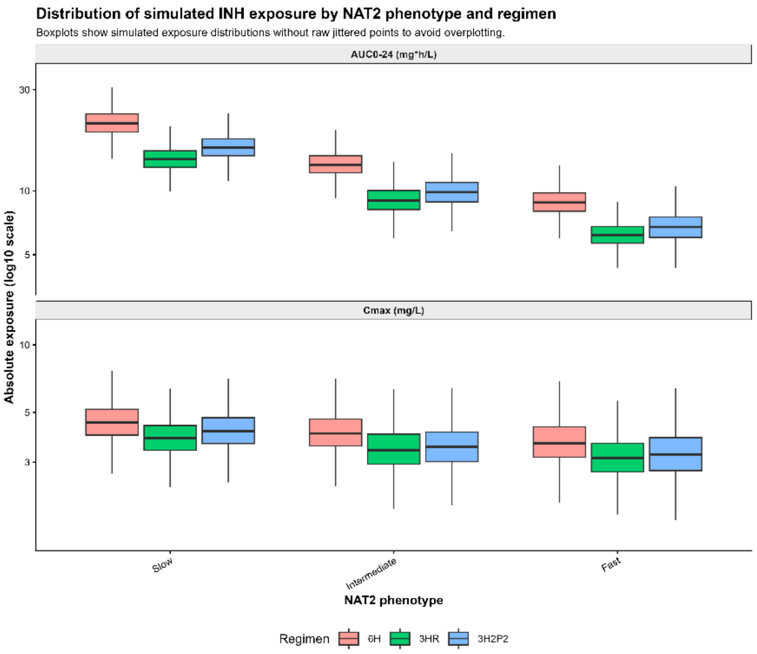

Supplementary Figure S2. Distribution of model-simulated isoniazid (INH) exposure (AUC_{0-24}, mg·h/L) by NAT2 acetylator phenotype and treatment regimen. Boxplots summarize the posterior predictive distributions of area under the concentration–time curve from 0 to 24 h (AUC_{0-24}) for each regimen (6H, 3HR, $3H_2P_2$) and NAT2 phenotype (slow, intermediate, fast). Simulations were performed using the final Bayesian population pharmacokinetic model, incorporating inter-individual variability and covariate effects. The lower and upper hinges of each box correspond to the 25th and 75th percentiles, with the median indicated by the central line. Whiskers extend to the most extreme data points within 1.5 times the interquartile range. Individual raw data points are not shown (jitter omitted) to avoid overplotting and improve clarity. This figure complements Table 4 and Supplementary Table S2 by visualizing the full distributional separation of INH exposure across phenotypes and regimens. Abbreviations: AUC_{0-24}, area under the concentration–time curve from 0 to 24 h; INH, isoniazid; NAT2, N-acetyltransferase 2; 6H, 6 months of daily INH; 3HR, 3 months of daily INH plus rifampicin; $3H_2P_2$, 3 months of twice-weekly INH plus rifapentine.
